# Yellow Fever Useful

**Published:** 1859-08

**Authors:** 


*Yellow Fever Useful.*—The old adage is, that all things are made for some wise purpose. Until lately we have been at a loss to divine the virtue of Yellow Jack. But we have found it out. He comes to free our city of the hordes of quacks, in the shape of Homœopaths, Hydropaths, Corn Doctors, Pile Doctors, *et id omne genus.* As soon as our warm June sun begins to shine between the shoulders of these gentry, they have loud calls northward. Their lives are too precious to be thrown into a yellow-fever grave. At any rate, we get rid of them for several months in the year.—*New Orleans Medical News and Hospital Gazette.*

The above shows, indeed, that yellow fever serves one good purpose; and if we can form any idea of the sensitiveness of quackdom on the subject of this epidemic, from their behavior in cholera seasons, we can easily imagine that there are some months in the year when New Orleans is tolerably free from charlatans. The unpaid, unthanked, regular profession generally bear the brunt of epidemics; and quacks, in taking prudent precaution to preserve their own lives, leave for a season; but, alas! only for a season.

